# Prevalence of accessory deep peroneal nerve in referred patients to an electrodiagnostic medicine clinic

**DOI:** 10.1186/1749-7221-6-3

**Published:** 2011-07-08

**Authors:** Seyed Mansoor Rayegani, Elham Daneshtalab, Mohamad Hasan Bahrami, Dariush Eliaspour, Seyed Ahmad Raeissadat, Sajjad Rezaei, Marzieh Babaee

**Affiliations:** 1 Physical Medicine and Rehabilitation Research Center, Shahid Beheshti Medical University, Tehran, Iran; 2 Department of Physical Medicine and Rehabilitation, Shohada Medical Center, Tehran, Iran

## Abstract

**Background:**

Accessory Deep Peroneal Nerve (ADPN) is an anatomic variation that can potentially cause disturbance in electrodiagnostic studies. This anomaly could be detected by nerve conduction studies. There are no recent updates about prevalence of this anatomic variation. Electrodiagnostic medicine clinic is the best environment for detecting presence and prevalence of this nerve, so present study enrolled.

**Materials & Methods:**

In this cross sectional descriptive study that take place from March 2009 to July 2010, 230 cases comprising 460 legs referred for electrodiagnostic studies of upper limbs problems participated in the study. Compound muscle action potential (CMAP) and Nerve conduction Velocity (NCV) of Deep Peroneal Nerve (DPN) were measured by using EMG machine by stimulating DPN at knee, ankle and lateral malleolous areas accordingly, with recording from extensor digitorum brevis muscle. Results were analyzed and conclusion made.

**Results:**

The study population included 120 females (52%) and 110 (47%) males with mean age of 42.1 ± 13.5 years. ADPN was detected in 28 patients (12%). Among them,10(17.9%) had bilateral ADPN and in remained 18 cases (82.1%) APN was unilateral. In 8 patients there was no recorded CMAP from EDB by proximal and distal stimulation implying EDB agenesis. Gender distribution was similar which means half of the cases (14 patients) belonged to each gender.

**Conclusion:**

The prevalence of ADPN in this study was 12.2%, (17.9% bilateral and 82.1% unilateral).

## Introduction

**Figure 1:**
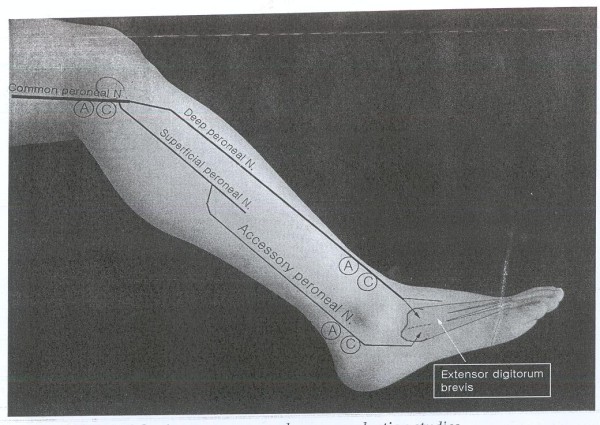
**Anatomy of ADPN**. This figure shows graphic anatomic course of ADPN.

Accessory Deep Peroneal Nerve (ADPN) is an anatomic variation which can potentially disturb electrodaignostic studies [1,2]. This nerve is separated from Superficial Peroneal Nerve (SPN) and then turns around lateral malleolus to innervate all or part of Extensor Digitorum Brevis(EDB)[3,4] (figure 1).

If this anomaly exists among patients with peroneal nerve lesion, atypical presentations could be seen in their electrodiagnostic study (EDX) [5]. In case of Deep Peronel Nerve (DPN) lesion, denervation in all of DPN innervated muscles could be seen except for EDB [2,4]. In case of Superficial Peroneal Nerve (SPN) lesion at distal part of leg there is only impaired sensory SPN response, however in case of APN variation, denervation of EDB is also present. In addition, lesions in proximal part of SPN causes usually denervation in peroneus longus and peroneus brevis muscles plus finding of impaired sensory response of SPN. In case of APN these findings are accompanied by denervation potentials in EDB [6,7]. The above mentioned findings that are consequence of presence of ADPN could errornously label complete type of Deep Peroneal Nerve lesion as incomplete one and also in case of pure Superficial Peroneal nerve lesion as Common Peroneal Nerve lesion.

This anomaly should be considered whenever there is peroneal nerve injury with above mentioned unusual presentations [8]. Anomaly is suspected whenever eliciting the CMAP (Compound Muscle Action Potential) response of EDB following peroneal nerve stimulation at anterior of ankle would result either in small or no response [8,9]. Lack of denervation in EDB in case of DPN injury at ankle is also seen in this anatomic variation [10]. According to different studies, ADPN prevalence is estimated to be 13 to 25 percent [6,11]. Our study goal is to determine the prevalence of this nerve variation in our population and reemphasizing attention to this anatomic variation in routine electrodiagnostic studies.

## Methods & Materials

**Figure 2:**
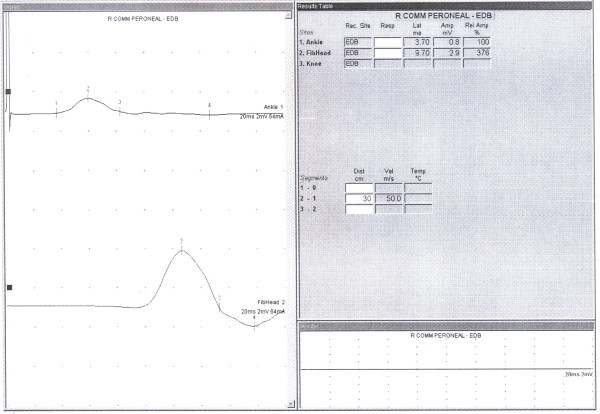
**NCS record of incomplete ADPN**. this graph is showing characters of CMAP record of incomplete type of ADPN.

**Figure 3:**
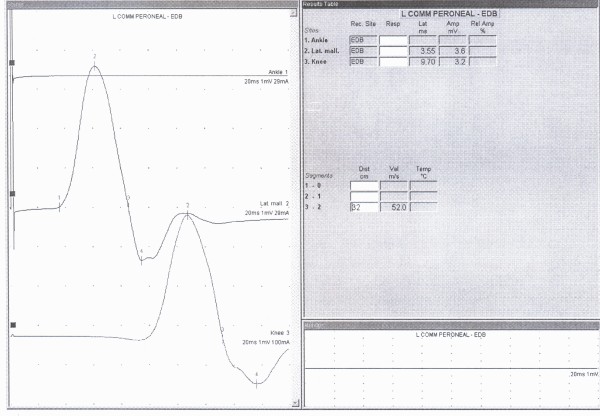
**NCS record of complete ADPN**. this graph is showing characters of CMAP record of complete type of ADPN.

In this descriptive cross sectional study 230 referred patients (460 legs) that was referred for EDX evaluation of upper limbs problems in Shohada EDX center, located at department of Physical medicine and Rehabilitation, after explaining the procedure and taking verbal consent were enrolled in the study. Lack of neurological complaints and normal neurologic examination of lower limbs were inclusion criteria for the cases. CMAP responses of DPN by proximal and distal stimulation were recorded from EDB muscle. Surface stimulation electrode using constant current was used for stimulation and surface bar recording electrode for recording. Proximal stimulation at knee region around fibular head and recording the response via EDB was done at first, distal stimulation was done at anterior of ankle and then shifted to lateral malleolus, while recording site was the same i.e EDB muscle. All responses were saved for analysis(figure 1). In cases in which CMAP responses were not recorded by distal (ankle) stimulation (figure 2), and/or its amplitude was lesser than proximal stimulation (figure 3) and instead by lateral malleolus stimulation the response was elicited, presence of APN was confirmed. In conditions that there was no recorded CMAP from EDB by proximal and distal stimulations (ant. of ankle and lateral malleolus), concentric EMG needle was used for recording of the CMAP response. Absence of the response even by this technique and lack of EMG activity by requesting patients to extend their toes at metatarsophalangeal joints without any denervation potentials, were assumed for agenesis of EDB. All tests were performed or directly supervised by a physiatrist attending. Toennis Neuro-screen model EMG machine was used for the study. Information including age, gender, and results of Nerve Conduction Velocity (NCV), Latency, CMAP of proximal and distal stimulation were recorded for analysis.

## Results

**Figure 4:**
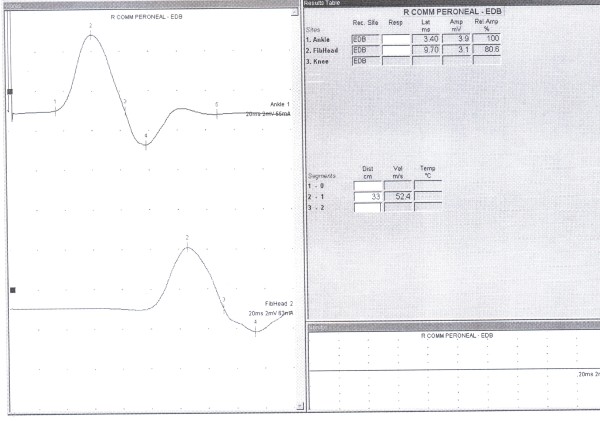
**NCS record of DPN without ADPN**. this graph is showing characters of CMAP record of DPN without ADPN.

120 females (52%) and 110 males (48%) with mean age of 42.11 ± 13 years (ranging from 15 to 65 years) were participated in the study. Mean of NCV, and CMAP amplitude by distal and proximal stimulation of DPN in patients without ADPN were recorded and analyzed. (Additional file 1: Table S1 and Figure 4). 28 patients (12%) including 14 male and 14 female were detected to have ADPN. 10 patients had bilateral and remained 18 patients unilateral ADPN. 8 patients had complete ADPN without recorded response from EDB by ankle stimulation and remained 20 patients had incomplete type of ADPN with recording the response from EDB by both ankle and lateral malleolus stimulation. (Additional file 2: Graph 1)

In 8 patients there were no recorded EDB responses by proximal and distal stimulation via surface and needle recording and there was no EMG activity and palpable EDB muscle mass by voluntary extension of toes at metatarsophalangeal joints.

## Discussion

Presence of Accessory Deep Peroneal Nerve (ADPN) may complicate electrodiagnostic and clinical features of peroneal nerve palsy including Common Peroneal, Deep Peroneal & Superficial peroneal parts. So that presence of ADPN could errornously label complete type of Deep Peroneal Nerve lesion as incomplete one and also in case of pure Superficial Peroneal nerve lesion as Common Peroneal Nerve lesion.

ADPN is a branch of superficial peroneal nerve, that has sensory and motor branches. The sensory branch innervates ankle joint, tendons and ligaments, and motor branch to peroneus longus and EDB [4]. The nerve can sometimes be entrapped at ankle and cause pain and discomfort at foot and ankle [11].

Our results indicate that, prevalence of ADPN is 12%. We found that 35% of 28 patients (10 patients) had bilateral type of ADPN. In our study there is no difference between female and male in distribution of APN. Other issue that was detected and explained in our study is detection of EDB agenesis that was not shown and mentioned in other studies [2,12].

Prevalence of ADPN was calculated up to 28% in other studies [13,14]. Mathis and et al. according to electrophysiological study of DPN and APN in 200 healthy subjects(400legs) shown 13.5% prevalence of APN. This finding is similar to our results [11]. Koudah and et al. studied the presence of accessory deep peroneal nerve on Japanese cadavers and reported that it was consistently present in 100% of speciments [15]. But Hasegawa et al. based on electrophysiological studies in Japanese persons demonstrated 17-28% prevalence of accessory deep peroneal nerve [16]. Bhardwaj and et al. shown 33.3% prevalence of ADPN in Indian cadavers [12]. This difference could be due to some genetic issues and/or techniques used that needs further studies to be confirmed. Also Kayal and et al. described this difference" the reported evidence is higher in studies that routinely applied stimulation behind the lateral malleolus during the performance of peroneal motor conduction studies" [2].

Marciniak study indicated 74% of patients had bilateral type of APN [17], which is more than our result(35%). Bilateral ADPN in this study was higher in males while in another studies it was higher in females [10,18]. No difference was found in our study.

Owaski and et al described the higher prevalence of APN in relative of healthy control group, reflecting autosomal dominant transmission [6]. Studies show that, the inheritance pattern of the accessory deep peroneal nerve is an autosomal dominant trait [7].

The most important parameter for detecting ADPN is comparing distal to proximal CMAP of EDB. In cases that distal CMAP was smaller than proximal stimulation, and/or no CMAP detected by distal "ankle" stimulation, this variation should be suspected. Mathis and et al. explained same result. They compared electrophysiological parameters in patients with and without ADPN. Their study showed a significant higher DPN motor potential area ratio (distal/proximal ratio) in subjects without APN [11].

The finding of no recorded EDB CMAP by distal and proximal stimulation of DPN and also lack of EMG activities and palpable EDB muscle mass by voluntary extension of toes at metatarsophalangeal joints, that was detected in 8 cases, was assumed as agenesis of EDB. This was not mentioned it in other studies. Owsiak and et al.found that, ADPN may innervate greater part of the EDB[6]. Ubogu explained about complete innervations of EDB by APN in his study without mention to the possibility of EDB agenesis [13].

## Conclusion

This study demonstrated that ADPN prevalence in referred patients for electrodiagnostic study of upper limbs to our clinic was 12%. There were also 8 patients with signs of EDB agenesis. This study showed again the significant prevalence of this anatomic variation and its potential role in complicating the electrodiagnostic interpretation of peroneal nerve study. There is no difference between male and female in our study.

## Competing interests

The authors declare that they have no competing interests.

## Authors' contributions

SMR proposed the topic of study, performed some of tests, designed the article, translated to English and supervise the work. ED performed some of tests, prepared the figures. MHB performed some of tests, reviewed articles. DE performed some of tests, SAR performed some of tests. SR participated in the design of the study. MB participated in the design of the study. All authors read and approved the final manuscript.

## Supplementary Material

Additional file 1 **Table S1: NCS of cases without ADPN**. presentation of NCS of cases without ADPN by NCV, amplitude and gender distribution. Click here for file

Additional file 2 **graph 1: total numbers of cases**. all cases are shown in graphic presentation including total, with and without ADPN and EDB agenesis Click here for file
